# The Ability of Resveratrol to Attenuate Ovalbumin-Mediated Allergic Asthma Is Associated With Changes in Microbiota Involving the Gut-Lung Axis, Enhanced Barrier Function and Decreased Inflammation in the Lungs

**DOI:** 10.3389/fimmu.2022.805770

**Published:** 2022-02-21

**Authors:** Esraah Alharris, Amira Mohammed, Hasan Alghetaa, Juhua Zhou, Mitzi Nagarkatti, Prakash Nagarkatti

**Affiliations:** Department of Pathology, Microbiology and Immunology, School of Medicine, University of South Carolina, Columbia, SC, United States

**Keywords:** resveratrol, asthma, ovalbumin, lung microbiome, gut microbiome

## Abstract

Asthma is a chronic respiratory disease highly prevalent worldwide. Recent studies have suggested a role for microbiome-associated gut–lung axis in asthma development. In the current study, we investigated if Resveratrol (RES), a plant-based polyphenol, can attenuate ovalbumin (OVA)-induced murine allergic asthma, and if so, the role of microbiome in the gut–lung axis in this process. We found that RES attenuated allergic asthma with significant improvements in pulmonary functions in OVA-exposed mice when tested using plethysmography for frequency (F), mean volume (MV), specific airway resistance (sRaw), and delay time(dT). RES treatment also suppressed inflammatory cytokines in the lungs. RES modulated lung microbiota and caused an abundance of A*kkermansia muciniphila* accompanied by a reduction of LPS biosynthesis in OVA-treated mice. Furthermore, RES also altered gut microbiota and induced enrichment of *Bacteroides acidifaciens* significantly in the colon accompanied by an increase in butyric acid concentration in the colonic contents from OVA-treated mice. Additionally, RES caused significant increases in tight junction proteins and decreased mucin (Muc5ac) in the pulmonary epithelium of OVA-treated mice. Our results demonstrated that RES may attenuate asthma by inducing beneficial microbiota in the gut-lung axis and through the promotion of normal barrier functions of the lung.

## Introduction

Asthma is an incurable disease (1). It is one of the most common chronic respiratory diseases seen globally. Currently, it affects over 300 million people globally and its incidence continues to grow (2). It is also estimated that the global medical costs of treating asthma is as high as $100 billion per year (3). Asthma is now considered to be a heterogeneous disease with multiple immunological pathways involved. However, the well-characterized pathway includes the activation of excessive T-helper type 2 (Th2) cells along the bronchial airways and massive production of pro-inflammatory Th2 cytokines such as IL-4, IL-5 and IL-13 (4). The release of Th2 cytokines during an asthma attack initiates a cascade of inflammatory responses along the barrier sites of the pulmonary system (4, 5).

Lungs are lined by epithelial cells that come in contact with environmental factors and immune cells. Thus, epithelium along with the immune cells plays the critical barrier function against external antigens. Together with the cellular immune system, the epithelium performs a pivotal role as the first line physical barrier against external antigens (6). Paracellular space is sealed by the tight junction proteins and therefore any dysfunction in the tight junction increases paracellular permeability, and immune cell activation and contributes to the pathogenesis of chronic lung inflammation (6). Epithelial cell disturbance involving tight junction can also activate Th2 cells and promote allergic asthma (6). Excessive inflammation at the bronchial epithelium can cause additional disruption of tight junction molecules, further compromising its barrier integrity. Such structural changes are referred to as airway remodeling and in asthma, it can lead to subepithelial fibrosis, increased smooth muscle mass, gland enlargement, mucus hypersecretion, neovascularization, and epithelial alterations (7).

Resveratrol (RES), a plant-derived bioactive polyphenol, widely studied and commercially available as a dietary supplement, has been proven effective in the attenuation of inflammatory responses in a variety of disorders due to its anti-inflammatory, anti-oxidative, and anti-microbial properties (8–10). RES acts through a variety of immunomodulatory pathways to suppress inflammation (11), including the induction of Tregs to suppress the effector functions of pro-inflammatory lymphocytes (12, 13). RES also possessed the ability to affect lung function directly by modulating microbial composition through its antimicrobial activity, leading to the alteration in the profile of circulating metabolites produced by commensal organisms (14–16).

Lung and gut microbiota play a critical role in the maintenance of healthy immune response. Thus, dysbiosis and subsequent dysregulation of microbiota have been linked to numerous clinical disorders, including asthma (17, 18). The concept of the gut-lung connection arose when it was noted that different types of diseases of the lung can be influenced by intestinal microbiota (18). Similarly, intestinal complications have also been noted during respiratory disease (18). The existence of such gut-lung axis has raised the possibility that agents that attenuate allergic asthma may do so through regulation of microbiota in the gut-lung axis, a concept that needs further evalution. 

Previous studies from our laboratory demonstrated that RES attenuates a variety of inflammatory disorders by inducing beneficial microbiota that promotes anti-inflammatory Tregs while suppressing pro-inflammatory Th1/Th17 cells (19–21). Additionally, we and others have shown that RES can attenuate allergic asthma-induced inflammation and remodeling (22–26). However, whether the ability of RES to attenuate allergic asthma is also related to potential alterations that RES can induce in the microbiota in the gut-lung axis has not been previously investigated. In the current study, therefore, we evaluated the effect of RES in the regulation of pulmonary and gut microbiota and investigated if such alterations induced beneficial bacteria that would help suppress inflammation and remodeling in Ovalbumin (OVA)-induced allergic asthma.

Many experimental animal models have been established to mimic human respiratory asthma. One of the most widely used mouse models for human asthma is the use of OVA in BALB/c mice (27). OVA challenged BALB/c mice shows robust airway hyper-responsiveness, goblet cell hyperplasia, epithelial cell hypertrophy and a strong Th2 response with the induction of IL-4 and IL-13 (28). Using this model, in the current study, we investigated if RES attenuates OVA-induced asthma, and if this is associated with significant alterations in the microbiota found in the lungs and the gut.

## Materials and Methods

### Animal Treatment

Female BALB/c mice, aged 6-8 weeks were purchased from the Jackson Laboratories (Bar Harbor, ME) and housed in specific pathogen-free conditions at the University of South Carolina School of Medicine Animal Facilities. The mice were maintained under a 12 h light/dark cycle at an ambient temperature of 24 ± 1°C in a specific pathogen-free animal facility and received *ad libitum* access to normal chow diet and water, as described (26). The allergic asthma was induced as described previously (26). Briefly, mice were divided randomly into three groups (Naïve, Ova-veh and Ova-res) and kept in isolated cages. After one week of housing, each mouse received an intraperitoneal (i.p.) injection of 100 µl sterile phosphate-buffered saline (PBS) solution containing 250µg chicken egg ovalbumin (OVA) (Sigma-Aldrich, USA) and 4mg/ml aluminum hydroxide on day 0. On day 7, the sensitized mice were challenged with 50 μg OVA suspended in 50 μl of sterile PBS intranasally under short anesthesia, as described (26). On days 1-14, mice in the treatment group (Ova-res) were administered daily with oral gavage of 200µl carboxymethyl cellulose (CMC) solution containing RES (100mg/kg) while the vehicle group (Ova-veh) received 200µl CMC solution alone. We chose the dose (100 mg/kg body weight) of RES based on our previous studies in which we found this dose to be optimum in attenuating allergic asthma (26). The mice were euthanized on day 15 under anesthesia for collection of serum samples, bronchoalveolar lavage fluids (BALF), lung tissues, and gut content.

### Plethysmography Assessment of Airway Hyperreactivity

Pulmonary function tests (PFT) in response to asthma induction were evaluated using 4-chamber, whole-body plethysmography (Buxco, Troy, NY), as previously described (10, 21, 29, 30). The respiratory flow was measured by a pneumotachograph and transmitted to Buxco FinePointe software for functional analysis to calculate the respiratory rate (frequency), lung volume, lung tissue resistance to flow, peak inspiratory and expiratory flow, and time intervals between breaths. Four mice were randomly selected from each experimental group and acclimated in Buxco chamber for 10 minutes. Baseline (BL) airway functionality levels were collected following the initial acclimation period for 5 minutes. Following baseline measurements, mice were exposed to aerosolized PBS for 30 seconds and then recorded for 3 minutes. Next, the mice were exposed to nebulized 25 mg/ml methacholine (Mch) for 30 seconds to induce bronchoconstriction and then recorded for 3 minutes. From the beginning to the end of the PFT evaluation, different parameters were evaluated and recorded every 2 seconds (30).

### Gut Microbiota Profiling

Previous studies have tested colonic content to study the microbiota and demonstrated that during Ova-induced asthma, significant dysbiosis occurs in the colon (31). Additionally, the short-chain fatty acids (SCFA) that are known to suppress asthma are produced in the large intestine (32). In the current study, we therefore tested the colonic content of mice for microbiota. To that end, colon flush was performed of the excised whole colon using sterile PBS, as described (19, 20). Bacterial genomic DNA was isolated from colon contents using QIAamp DNA Stool Mini Kit (Qiagen, Valencia, CA) and quantified using a Qubit 4 Fluorometer (ThermoFisher, Waltham, MA). Purified DNA was used in the library preparation for 16S ribosomal RNA sequencing using 16S Metagenomic Sequencing Library Preparation Kit (Illumina, San Diego, CA) according to the manufacturer’s instructions, and as detailed elsewhere (20, 33, 34). Briefly, the purified DNA was amplified by PCR using bacterial/archaeal sense 319 F primer and anti-sense 806 R primer to amplify the V3-V4 hypervariable regions of 16S rRNA. After clean-up, the amplified PCR products were then amplified again by PCR using dual indices and sequencing adaptors for 16S rRNA sequencing (35, 36).

### Lung Microbiota Profiling

The lung microbiota profile was conducted as described elsewhere (19, 20, 34). Quick-DNA Fungal/Bacterial Midiprep Kit (Zymo Research Corp, Irvine, CA) was used in the isolation of bacterial genomic DNAs from lungs according to the manufacturer’s instructions. Briefly, 500 mg of lung tissue was added to ZR Bashing/Bead lysis filtration tube with 6 ml of genomic lysis buffer containing β-mercaptoethanol (0.5% v/v). The tubes were placed in a 50ml–tube holder for 1 minute, followed by centrifugation at 3,000 g for 5 minutes. Following centrifugation, the samples were filtered through Zymo-Spin V-E columns in a vacuum manifold with a vacuum pressure of 600 mmHg. After filtering, the columns were transferred to collection tubes for centrifugation at 10,000 g for 1 minute. The columns were washed with 400 µl of genomic DNA-wash buffer and then DNA samples were eluted from the columns with 150 µl DNA Elution Buffer under centrifugation at 10,000 g for 1 minute. DNA samples were stored at -80°C and used in the library preparation for 16S ribosomal RNA sequencing using 16S Metagenomic Sequencing Library Preparation Kit (Illumina, San Diego, CA) according to the manufacturer’s instructions.

### Detection of Lipopolysaccharide (LPS) in BALF

Bronchoalveolar lavage fluid (BALF) samples were collected from mice as described previously (25). Concentrations of LPS in BALF samples from mice were detected using Pierce LAL Chromogenic Endotoxin Quantitation Kit (Thermo Fisher Scientific, Waltham, MA) according to the manufacturer’s protocol (36).

### Detection of Cytokines and Chemokines in BALF, Serum, and Culture Supernatants

The concentrations of cytokines and chemokines in BALF, serum, and culture supernatants were measured as described earlier (10, 21, 25). To generate culture supernatants, lung-infiltrating mononuclear cells were isolated as described later and cultured in tissue culture medium (DMEM) for 24 hours. The culture supernatants were then tested for cytokines. The cytokines were detected using ELISA kits according to the manufacturer’s protocols. IL-1β, IL-4, IL-6, IL-13, IFN-γ, and TNF-α ELISA kits were purchased from Biolegend (San Diego, CA), whereas IL-10 and TGF-β ELISA kits were purchased from R&D Systems (Minneapolis, MN).

### Immunofluorescence Analysis of Epithelial Mucin

Mucin expression in lung epithelial cells was detected by immunofluorescence assay using anti-mucin 5AC monoclonal antibody (Abcam, Cambridge, MA). Briefly, lung tissues were fixed with 4% paraformaldehyde (MilliporeSigma, St. Louis, MO) overnight and embedded in paraffin. Five-micron sections were obtained and transferred onto the slides. The slides were then dewaxed and rehydrated with gradually decreasing ethanol gradient solutions followed by PBS. The slides were treated with antigen retrieval solution (100 mM Tris, 5% urea, pH=9.5) in a 95°C water bath for 10 min and then with permeabilization buffer (0.1M triton, 0.01M glycine in PBS) for 30 min. After washing with PBS three times, the slides were treated with the blocking solution [5% goat serum and 1% bovine serum albumin (BSA) in PBS] for 30 minutes. After washing, the slides were incubated with a primary anti-mouse mucin 5AC antibody at 4°C overnight. On the next day, the slides were washed with PBS and incubated with the secondary Alexa Flour-488 conjugated goat anti-mouse antibody (Abcam, Cambridge, MA) diluted in PBS with 1% BSA (1:1000) in a 37°C incubator for 2 h. After washing with PBS, the slides were counterstained with DAPI (1:5000) (Thermo Fisher Scientific, Waltham, MA) on a shaker for 20 min. After washing, the slides were mounted with Prolong Diamond Antifade Mountant (Thermo Fisher Scientific, Waltham, MA), examined and photographed under Leica immunofluorescence microscope.

### Real-Time Quantitative PCR (RT-qPCR) of Gene Expression

Pulmonary epithelial cells were isolated as described previously (13). Briefly, euthanized mice were subjected to lung perfusion by three lung lavages with cold sterile PBS using a 20-gauge catheter. A separate catheter was used to inject 1ml of dispase solution at the concentration of 25 U/ml into the lungs for 30 sec and the lungs were washed by three lavages with cold sterile PBS. Next, the lungs were harvested for physical disruption. Each lung lobe was cut into small pieces and transferred into a 50 ml tube containing 25 U/ml dispase on a shaker for 45 minutes. After dispase digestion, the samples were combined with 10 ml of sorting buffer (2% fetal bovine serum (FBS) and 50 U/ml DNase in DMEM) and then incubated at 37°C for 10 min while shaking. After incubation, the samples were filtered through cell strainers with pore sizes of 100µm, 70µm, and 40µm, respectively. After the triple filtrations, the samples were transferred to 15 ml tubes and centrifuged at 550 g for 5 min at 4°C. After centrifugation, the cell pellets were suspended in 10 ml of sorting buffer and incubated at 37°C for 1 h while shaking. After incubation, the samples were spun down at 550 g at 4°C for 8 min and re-suspended in 700µl QIAzol lysis reagent each sample for RNA isolation from lung epithelial cells using RNeasy Mini Kit (Qiagen, Germantown, MD).

Pulmonary infiltrating cells were isolated as described previously (13, 37, 38). Briefly, lung tissues were collected from euthanized mice and infused with cold sterile PBS. Lung tissues were dissociated using a stomacher and then treated with red blood cell (RBC) lysis buffer at a concentration of 250µg/ml for one min. Ten mL of FACS buffer (2% FBS in PBS) was added to neutralize RBC lysis buffer and the samples were then centrifuged at 300 g at 4°C for 10 min. After centrifugation, the cell pellets were suspended in 5 ml of FACS buffer and then slowly added on the top of 5mL Ficoll-Paque (Thermo Fisher Scientific, Waltham, MA). The mixtures were centrifuged at 500 g for 30 min at room temperature. After centrifugation, the interphase representing the infiltrating mononuclear cells was then saved and suspended in 10 ml of FACS buffer. After centrifugation, the cell pellets were re-suspended in 700µl QIAzol lysis reagent for RNA isolation from pulmonary infiltrating cells using RNeasy Mini Kit (Qiagen, Germantown, MD).

cDNA was synthesized from total RNA samples from the pulmonary epithelial cells and infiltrating cells using Bio-Rad miScript Kit according to the manufacturer’s protocol (Bio-Rad Laboratories, Hercules, CA). RT-qPCR was conducted on the generated cDNA samples for detection of gene expression including Cadherin-1, Claudin-18, ZO-1, and Occludin. The expression levels were normalized to GAPDH. The sequences of primers for RT-qPCR are shown in **Supplemental Table 1**.

### Identification and Quantification of SCFAs

SCFAs were detected as described (19). Briefly, colonic contents were suspended in water. After centrifugation, the supernatants were saved and acidified by HCl. 4-methyl valeric acid was used as an internal standard. SCFAs were identified and quantified by the gas chromatograph CP-3800 (Varian) and mass spectrometry (GC-MS) system (39). Varian MS Workstation (version 6.9.2.) software was used to collect and analyze the data. The linear regression equation was used to calculate the concentrations of acetic acid and propionic acid in colon samples.

### MLE-15 Cell Culture and Evaluation of Their Response to LPS-Mediated Injury

MLE-15 murine lung epithelial cell line was purchased from Creative Bioarray (Shirley, NY) and cultured in a mixture of DMEM and Ham’s F12 medium (1:1) containing 10% FBS, 2% ITS (insulin, transferrin, and sodium-selenite, Sigma-Aldrich), 50µM hydro cortisol, 50µM estradiol and 1% penicillin/streptomycin. LPS (Invitrogen, Thermo Fisher Scientific, Waltham, MA) was used at a dose of 20ng/ml to induce epithelial injury and the cultures were incubated at 37°C in the presence of 50 µM of RES or VEH for 48 h. Then, the culture supernatants were collected, filtered with 0.2µm filters to obtain cell-free supernatant, and stored at -80°C for cytokine analysis. Cultured MLE-15 cells were collected by trypsin digestion and suspended in 700µl QIAzol lysis reagent for RNA isolation using RNeasy Mini Kit (Qiagen, Germantown, MD) (40).

### Transepithelial Electrical Resistance (TEER) Measurement of LPS-Injured MLE-15 Cells

MLE-15 cells (5×10^4^ per well) were seeded in 8-well array plates (Applied BioPhysics, New York, NY) and allowed to grow at 37°C with 5% CO2 in an incubator until confluence. When the cells reached confluence, 20ng/ml LPS-containing medium was added having 50µM RES (LPS+res) or VEH (LPS+veh) and resistance values (Ohm.cm^-1^) were recorded using ECISzθ system (Applied BioPhysics, New York, NY) every 5 min up to 48 h. Multi-current frequencies were also recorded by using multi-frequency test mode (MFT). Then the acquired data were analyzed to evaluate the effects of RES on the barrier function of LPS-injured epithelial cells (40).

### Statistical Analysis

In all experiments, we used groups of 5 mice based on power analysis, unless otherwise mentioned in the Figure legends. Also, the number of mice used in each experiment is detailed under the Figure legends. The experiments were repeated at least 3 times with consistent results. Data were presented as mean ± SEM. Differences among more than two groups were assessed using a one-way ANOVA test followed by Tukey *post hoc* test in Graph Prism V8.00 for Windows (San Diego, CA). Student’s t-test was used to compare the differences between two experimental groups. p<0.05 was considered to have a significant difference. Different significant levels were depicted as *p<0.05, **p<0.01, ***p<0.001 and #p<0.0001, in the figure legends.

## Results

### Resveratrol Attenuates Physiological and Histological Asthmatic Features in the Lungs Induced by OVA

**Figure 1:**
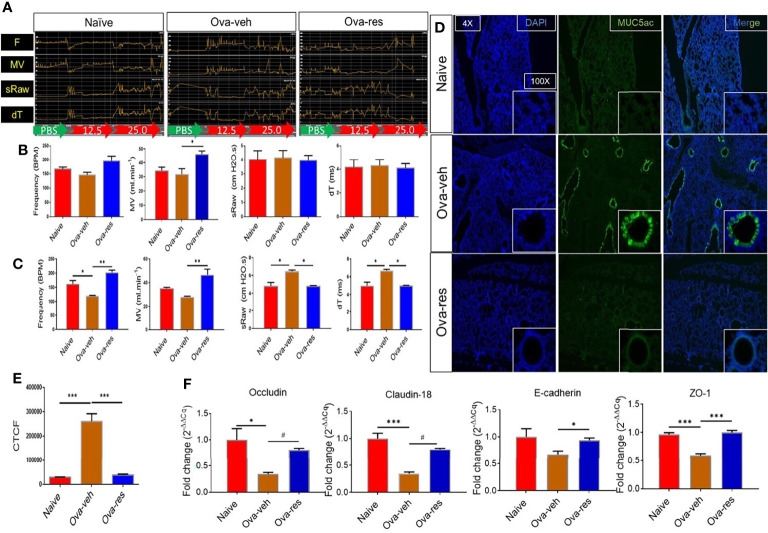
Effect of resveratrol on physiological functions of the lungs in OVA-administered mice. BALB/c mice were initially sensitized by intraperitoneal injection of ovalbumin followed by an intranasal challenge on day 7. These mice received vehicle (Ova-veh) or RES (Ova-res) on days 1-14. On day 15, the mice were chosen randomly and placed in one of 4 chambers of Buxco instrument for pulmonary function analysis or sacrificed for other examination. **(A)** Pulmonary function tests included changing patterns of frequency (F), mean volume (MV), specific airway resistance (sRaw) and delay time (dT) among naïve, Ova-veh and Ova-res group as baseline response (BL, green arrow), PBS exposure response (red arrow), and 25 mg/ml methacholine response (Mch, red arrow). Bar graphs present the statistical comparison of frequency, MV, sRaw and dT among naïve, Ova-veh and Ova-res group at the baseline response **(B)** and after challenge with 25 mg/mL methacholine **(C)**. **(D)** Immunofluorescent staining showing the expression of MUC5ac protein in lung sections of naïve, Ova-veh and Ova-res group mice observed under Leica microscope. Statistical comparison of MUC5ac protein expression among naïve, Ova-veh and Ova-res group **(E)**. RT-qPCR data to measure the expression of tight junction proteins, occludin, claudin-18, E-cadherin and ZO-1 in purified epithelial cells from mice **(F)**. Vertical bars in all panels represent Mean+/-SEM data from groups of 5 mice. *p<0.05, **p<0.01, ***p<0.001, ^#^p<0.0001.

We first investigated the effect of resveratrol on lung functions following induction of asthma by OVA. Pulmonary function tests included measurement of F (frequency), MV (mean volume), sRaw (specific airway resistance), and dT (delay time) among naïve, Ova-veh, and Ova-res group as baseline response (green arrow), PBS exposure (red arrow) and following methacholine (Mch) exposure (red arrow) (**Figure 1A**). **Figure 1A** shows a representative experiment and data from multiple experiments are plotted in **Figure 1B** (showing baseline response) and **Figure 1C** (showing after challenge with Mch). The data showed that the various parameters studied were not altered in the 3 groups at the basal level except the MV which was increased in the Ova-res group when compared to Ova-veh group (**Figure 1B**). However, in the Ova-veh group, there was a significant decrease in frequency, and an increase in sRaw and dT, and no significant change in MV when compared to the naïve group (**Figure 1C**). In contrast, the Ova-res group showed reversal of all the impaired lung functions (F, MV, sRaw, and dT) when compared to the Ova-veh group (**Figure 1C**). Additionally, we also studied changes in patterns of specific airway conductance (sGaw), peak of expiratory flow (PEF), time to inhale (Ti), and time to exhale (Te) among naïve, Ova-veh and Ova-res group **(** **Figure S1** **)** as baseline response (green arrow), PBS exposure (red arrow) and 5 mg/ml methacholine (Mch) exposure (red arrow) **(** **Figure S1A** **)**. The data showed that after Mch exposure, the Ova-veh group showed an increase in Te but not in other parameters when compared to the naïve group, however, Ova-res group showed increases in sGaw, PEF, and a decrease in Ti and Te when compared to the Ova-veh group **(** **Figure S1C** **)**. These data together suggested that the lung functions as measured by specific airway conductance, airway resistance, and time to inhale and exhale, significantly improved following treatment of mice exhibiting Ova-induced asthma, with resveratrol.

Because Muc5ac plays a key role in allergic inflammation and is necessary for airway hyperreactivity (AHR) to MCh (41), we next tested the effect of RES on MUC5ac expression. Immunofluorescent staining discovered that the production of epithelial MUC5ac protein was significantly increased in the lungs of OVA-veh-challenged mice when compared to the naïve mice (**Figures 1D, E** **)**, while RES was able to significantly decrease MUC5ac expression as measured by corrected total cell fluorescence (CTCF) when compared to Ova-veh group (**Figures 1D, E** **)**. Because asthma can also damage the airway physical barrier function that is regulated by tight junction (TJ) proteins, we investigated the TJ proteins. RT-qPCR analysis revealed that Ova-veh group showed a significant decrease in most of TJ proteins including occludin, claudin, and zonula occludens-1 (ZO-1) when compared to the Naïve group (**Figure 1F**), while Ova-res group showed a significant increase in occludin, claudin, E-cadherin, and ZO-1 when compared to the Ova-veh group (**Figure 1F**). These data suggested that while Ova caused increased mucus production and damage to the barrier function of the lungs, and resveratrol could reverse these effects.

#### The Beneficial Effect of Resveratrol on Lung Microbiota

**Figure 2:**
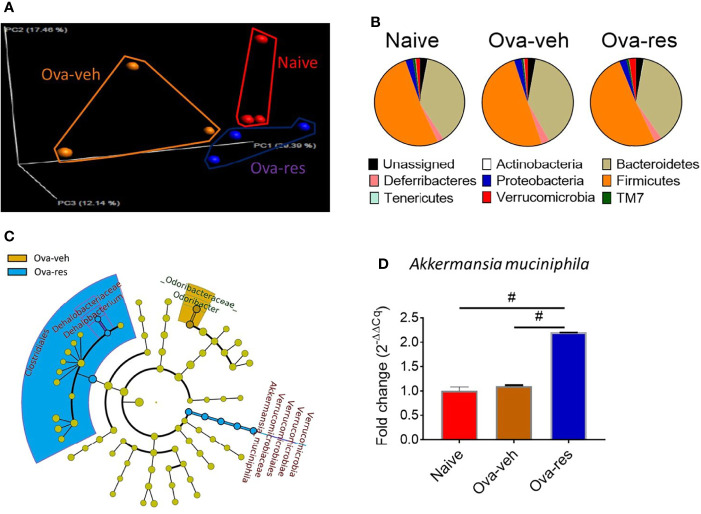
Effect of resveratrol on lung microbiota. OVA and RES treatments were performed as described in **Figure 1** legend. The lung microbiota profile was conducted as described in Methods. **(A)** Principal coordinator analysis showing the specific distribution of lung microbiota in naïve, Ova-veh and Ova-res groups. **(B)** Pie charts showing the percentages of common bacterial phyla in the lung microbiota among naïve, Ova-veh and Ova-res groups. **(C)** cladogram showing the least significant discriminative changes of lung microbiota between Ova-veh and Ova-res group with at least 2-fold changes. **(D)** qPCR showing the abundance of *Akkermansia muciniphila* in mouse lung microbiota among naïve, Ova-veh and Ova-res groups. Vertical bars in panel D represent Mean+/-SEM data from groups of 5 mice. In panel **(A)**, 2 mice out of 5 were excluded by Nephele setting). ^#^p<0.0001.

Because lung microbiota plays an important role in establishing immune system homeostasis in the lungs, next, we investigated if OVA caused any alterations in the lung microbiota and if RES had any impact. To that end, we used Nephele, an online platform for the analysis of microbiome data provided by the National Institutes of Health (NIH) to classify reads into operational taxonomic units (OTUs) and further analyze the 16S ribosomal RNA sequences generated from lung tissues (42). Principal coordinator analysis showed distinct clustering of mice among naïve, Ova-veh and Ova-res groups in their microbiota profile **(** **Figure 2A** **)**. Further analysis discovered that RES-treatment led to an increase in the abundance of the bacterial phylum Verrucomicrobia in lungs of OVA-challenged mice **(** **Figure 2B** **)** when compared to the other two groups. Also, the least discriminative analysis (LDA) was used to find out bacterial biomarkers and the cladogram showed that *Akkermansia muciniphila*, a species of Verrucomicrobia phylum, was significantly increased in RES-treated OVA-challenged mice, at least two folds higher than in Ova-veh or naïve group (**Figure 2C**). Quantitative PCR (qPCR) validation also confirmed the significant increase in the abundance of *A. muciniphila* in lung microbiota from Ova-res group when compared to naïve and Ova-veh groups (**Figure 2D**). These data suggested that RES could alter lung microbiota and increase the beneficial bacteria such as *A. muciniphila*.

#### Effect of Resveratrol on Gut Microbiota

**Figure 3:**
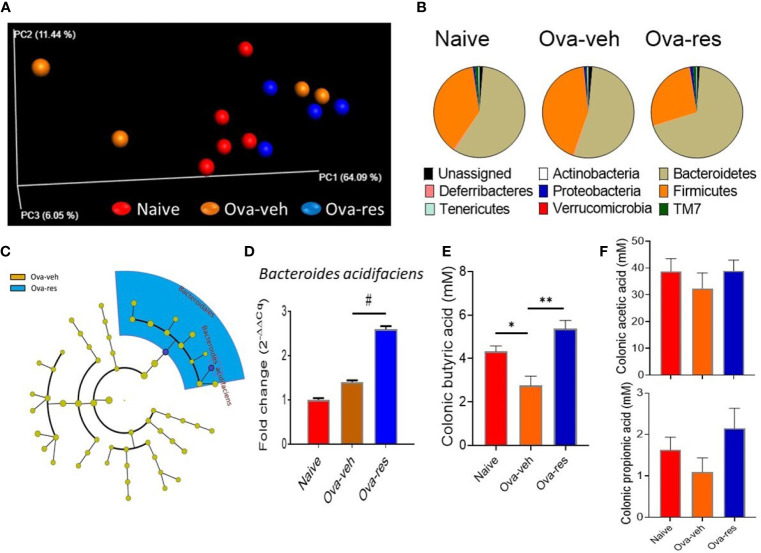
Effect of resveratrol on gut microbiota. The gut microbiota profile was conducted as described in Methods. **(A)** Principal coordinator analysis shows the specific distribution of gut microbiota in the mice belonging to the naïve, Ova-veh and Ova-res groups. **(B)** Pie charts showing the percentages of common bacterial phyla in the gut microbiota among naïve, Ova-veh and Ova-res groups. **(C)** Cladogram showing the least significant discriminative changes of gut microbiota between Ova-veh and Ova-res group with at least 2-fold changes. **(D)** qPCR was used to detect the abundance of *Bacteroides acidifaciens* in mouse gut microbiota among naïve, Ova-veh and Ova-res groups. Mass spectrometry–gas chromatography analysis showing the differences of butyric acid **(E)**, and acetic acid, and propionic acids **(F)** concentrations in mouse colonic materials among naïve, Ova-veh and Ova-res groups. Vertical bars represent Mean+/-SEM data from groups of 5 mice. *p<0.05, **p<0.01, ^#^p<0.0001.

Next, we tested if RES also caused alterations in the gut microbiota. 16s rRNA sequencing analysis displayed that there was no distinct clustering of groups based on the population of gut microbiota among naïve, Ova-veh and Ova-res groups (**Figure 3A**). However, RES treatment dramatically increased the abundance of the phylum Bacteroidetes but decreased the abundance of the phylum Firmicutes (**Figure 3B**). Furthermore, LefSe platform was used to analyze the LDA of gut microbial communities in Ova-induced mice and it was found that RES administration significantly increased the abundance of the order Bacteroidales and species *Bacteroides acidifaciens* in comparison with Ova-veh group (**Figure 3C**). qPCR confirmed that *B. acidifaciens* was significantly increased in Ova-res group in comparison to naïve and Ova-veh groups (**Figure 3D**). RES treatment significantly increased the concentrations of SCFA, butyric acid, in the colonic contents of OVA-injected mice (**Figure 3E**). However, RES did not significantly affect the production of propionic acid and acetic acid in mice **(** **Figure 3F** **)**. The results indicated that RES could also induce significant changes in the gut microbiota in asthmatic mice.

#### Effect of Resveratrol on LPS Metabolism

**Figure 4:**
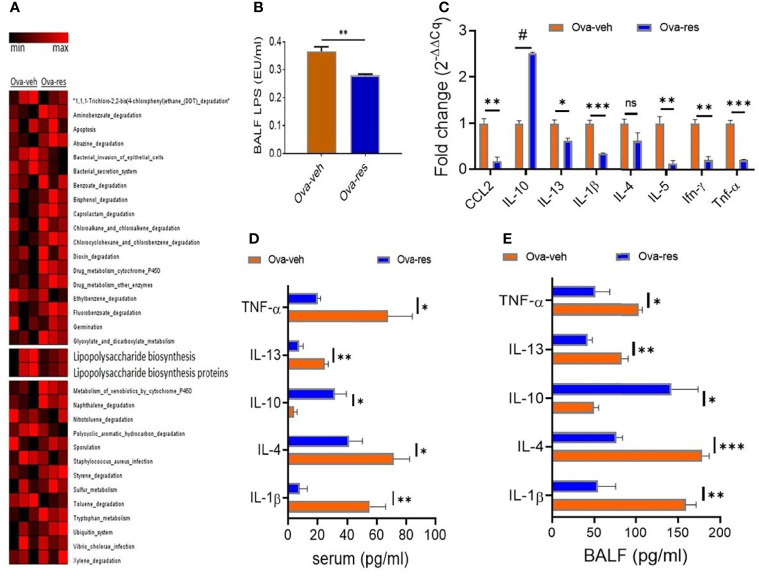
Effect of resveratrol on LPS metabolism and cytokine production. OVA and RES treatments were performed as described in **Figure 1** legend. **(A)** Heat map showing the predicted metagenomic functions of lung microbiota between Ova-veh and Ova-res group. The predicted biological functions were normalized to the mean OTUs of naïve group. **(B)** LPS concentrations in bronchoalveolar lavage fluid (BALF) were measured in Ova-veh and Ova-res groups. **(C)** RT-qPCR assay to measure gene expression of inflammatory and anti-inflammatory cytokines in the isolated mononuclear cells from mouse lungs of Ova-veh and Ova-res groups. ELISA measurements of different cytokine levels in the serum **(D)** and BALF **(E)** of OVA-exposed mice treated either with VEH or RES. Vertical bars in all panels represent Mean+/-SEM data from groups of 5 mice. In panel **(A)**, 1 mouse out of 5 in Ova-veh and Ova-res groups were excluded by Nephele setting). ns, not significant, *p<0.05, **p<0.01, ***p<0.001, ^#^p<0.0001.

Because our data showed RES could modulate lung microbiota in OVA-induced asthmatic mice (**Figure 2**) and LPS is one of the main components of gram-negative bacterial cell walls that drives inflammation, we investigated the effect of RES on LPS metabolism in mice. To that end, we investigated the functional profile of dysregulated lung microbiota in OVA-induced mice using a bioinformatics software package, Phylogenetic Investigation of Communities by Reconstruction of Unobserved States (PICRUST). Noticeably, we found that the microbial communities in the lungs of mice in Ova-veh group were highly involved in the biosynthesis of LPS, bacterial invasion of epithelial cells, bacterial secretion system, and *staphylococcus aureus* infection when compared to the lung microbiota of Ova-res group mice (**Figure 4A**). Interestingly, the LPS concentrations in mouse BALF were found to be significantly decreased in Ova-res group when compared to the Ova-veh group (**Figure 4B**). These results suggested that RES treatment may attenuate the levels of LPS production in OVA-induced asthmatic mice.

Additionally, when we tested the mice for cytokines and chemokines in the lung infiltrating mononuclear cells and found that there was a significant decrease in the induction of proinflammatory cytokines such as CCL-2, IL-13, IL-1β, IL-4, IL-5, IFN-γ, and TNF-α while noting a significant increase in anti-inflammatory cytokines such as IL-10 in the Ova-res group when compared to the Ova-veh group (**Figure 4C**). When we measured the cytokines in the serum and BALF, we found that TNF-α, IL-13, IL-4, and IL-1β were downregulated in mice treated with Ova+res when compared to the Ova-veh group (**Figures 4D, E** **)**. In contrast, anti-inflammatory cytokines such as IL-10 were increased both in the serum (**Figure 4D**) and BALF (**Figure 4E**) of Ova-res group when compared to Ova-veh group. These data suggested that resveratrol attenuates inflammatory cytokines while increasing the levels of anti-inflammatory cytokines in the lungs of mice with Ova-induced asthma.

#### Effect of Resveratrol on Integrity and Function of Epithelial Cells

**Figure 5:**
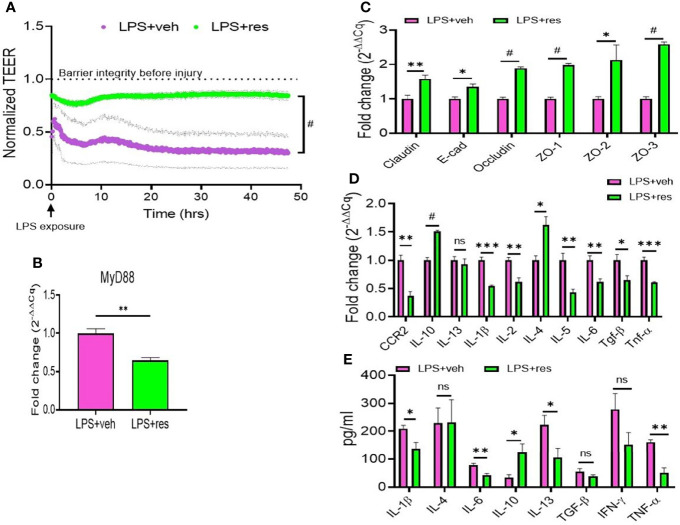
Effect of RES on the integrity and functions of mouse epithelial cells. MLE-15 mouse epithelial cell layer was exposed to LPS and treated with either RES or VEH. **(A)** Transepithelial electrical resistance (TEER) measurement system was used in the analysis of the integrity and permeability of cultured MLE-15 mouse epithelial cell layer exposed to LPS and treated with either RES or VEH. RT-qPCR was used to detect the gene expression in the LPS-treated MLE-15 cells incubated with RES or VEH. The genes studied included: **(B)** MyD88, **(C)** Tight junction proteins (claudin, E-Cad, occuludin, ZO-1, ZO-2 and ZO-3), **(D)** Cytokines and chemokines (CCR2, IL-10, IL-13, IL-1β, IL-2, IL-4, IL-4, IL-6, TGF-β and TNF-α). **(E)** ELISA was used in the measurement of cytokines (IL-1β, IL-4, IL-6, IL-10, IL-13, TGF-β, IFN-γ and TNF-α) in the supernatants of MLE-15 cell cultures in the presence of LPS and RES or VEH **(E)**. ns, not significant, *p<0.05, **p<0.01, ***p<0.001, ^#^p<0.0001.

To further understand the role of RES on lung epithelial cells, we used murine lung epithelial cell line, MLE-15, to study the effect of RES on the integrity and function of the alveolar epithelial cell layer. Our studies revealed that confluent MLE15 cell exposure to LPS led to the breaking down of epithelial cell barrier integrity as shown by the decrease in transepithelial electrical resistance (TEER) value of MLE-15 cell layer after LPS exposure (**Figure 5A**). However, RES treatment significantly increased the TEER value of MLE-15 cell layer in comparison with LPS+veh group, suggesting that RES could maintain the barrier integrity of the epithelial cell layer (**Figure 5A**). Also, RES treatment significantly inhibited the expression of MyD88 adapter gene in LPS-injured MLE-15 cells, indicating that RES may play an inhibitory role in the induction of the TLR4 signaling pathway triggered by LPS (**Figure 5B**).

Because RES could improve epithelial cell integrity and inhibit MyD88 (**Figures 5A, B**), we also examined the effects of RES on the expression of tight junction proteins and inflammatory cytokines in LPS-injured MLE-15 culture cells. Interestingly, we found that RES treatment significantly increased the expression of tight junction proteins such as claudin, E-cadherin (E-cad), occludin, ZO-1, ZO-2, and ZO-3 in LPS-injured MLE-15 cells as compared with the vehicle control cells (**Figure 5C**). In addition, RES treatment decreased the gene expression of pro-inflammatory cytokines including CCR2, IL-13, IL-1β, IL-2, IL-4, IL-5, IL-6, TGF-β, and TNF-α but increased the gene expression of anti-inflammatory IL-10 in LPS-injured MLE-15 cells (**Figure 5D**). ELISA analysis also confirmed that RES treatment decreased the production of pro-inflammatory cytokines such as IL-1β, IL-4, IL-6, IL-13, IFN-γ, and TNF-α while increasing IL-10 in the culture supernatants of LPS-injured MLE-15 cells **(** **Figure 5E** **)**. These results suggested that RES could increase the expression of tight junction proteins, and suppress pro-inflammatory cytokines, and promote anti-inflammatory cytokines following exposure of lung epithelial cells to LPS.

## Discussion

The current study demonstrates that RES can attenuate OVA-induced asthma in an animal model, consistent with previous studies (24–26). To test the effect of RES on asthma, lung functions were assessed using whole-body plethysmography at both the basal levels as well as following challenges with methacholine, as previously described (43, 44). The measurements of these parameters revealed that there was a deviance from normality in lung function in the Ova-veh group, characterized by a loss of general pulmonary functions. According to the output of clinical parameters, the Ova-res group exhibited restored lung function and displayed lung functionality similar to a normal state. This effect was observed with statistically significant improvement of F, MV, sRaw, and dT after RES treatment. For example, the specific airway conductance (sGaw) was significantly improved in Ova-res group when compared with Ova-veh group. Moreover, the parameters of specific airway resistance (sRaw) and delay time (dT) of moving the air between thoracic and nasal cavities were significantly improved in Ova-res group as compared with Ova-veh group when the animals were challenged with Mch. A reduction in sRaw in models of asthma, has often been used to indicate disease mitigation in response to drug treatment (44–47). The parameters of time to inhale (Ti) and time to exhale (Te) also decreased significantly in Ova-res group when compared to the Ova-veh group. These collective results showed that in an OVA-induced mouse model of asthma, RES attenuated the pathology associated with asthma and restored the normal lung functions.

OVA is well established to trigger Th2 cells producing IL-4, IL-5, and IL-13. Additionally, the epithelial cells can also produce many cytokines such as IL-1 IL-33, and TGF-β. The epithelial cells subsequently stimulate dendritic cells to produce a wide array of cytokines such as IL-1α, IL-1β, IL-6, IL-7, IL-12, IL-15, IL-18, TNF-α, TGF-β (48). Thus, asthma may involve a large number of proinflammatory cytokines and chemokines. In the current study, we noted that RES treatment was able to suppress several of the inflammatory cytokines both in the serum and the BALF as well as those produced by the isolated mononuclear cells from mouse lungs. In the current study, we also noted that RES caused significant induction of IL-10 which is produced by the regulatory T cells (Tregs) that play a crucial role in controlling asthma (49).

Previous studies have shown that exposure to airway irritants reduced the expression of tight junction molecules such as occludin and claudin within the bronchial epithelium, resulting in a lack of barrier integrity (50–52). Furthermore, the release of pro-inflammatory molecules during an asthmatic incident was found to disrupt tight junctions (48). Thus, we assessed mRNA levels of tight junction molecules and found that major components of epithelial tight junctions (claudin, cadherin, occludin, and ZO-1) were significantly higher in the Ova-res group when compared to the Ova-veh group. This finding suggested that RES may improve airway barrier integrity through the promotion of tight junction molecule expression, ultimately alleviating symptoms of asthma.

The role of the microbiota in the regulation of inflammation was highlighted by the proposed “hygiene hypothesis,” which suggested that the decreasing incidence of infections in western countries may play a role in increasing the incidence of both autoimmune and allergic diseases (53). The incidence of asthma, along with other atopic diseases, has increased over recent decades and was thought to be, at least partially, linked to reduced exposure to microbial agents during development (54). Several extrinsic factors experienced in early life, such as maternal administration of antibiotics during pregnancy, maternal exposure to pets, delivery by caesarian section, and feeding with infant formula are suggested to have lasting effects on the gut microbial constituents and were associated with the development of asthmatic symptoms (55). To further understand the role of microbiota in the underlying mechanisms through which RES alleviates asthma, the effect of RES on lung microbiota was investigated in the current study. Our analysis discovered that RES significantly increased the abundance of the phylum Verrucomicrobia and species *Akkermansia muciniphilia* in lung microbiota. *Akkermansia muciniphilia* is a gram negative, mucus-degrading bacterium that was considered beneficial when found at appropriate levels and has been implicated in a variety of inflammatory disorders (56). The increased colonization of *Akkermansia muciniphilia* within the gut epithelium mucosal layer was shown to combat metabolic diseases (57–59). The severity of asthma has been shown to negatively correlate with the levels of fecal *Akkermansia muciniphila* (60). Also, the administration of *Akkermansia muciniphila* into mice has been shown to attenuate airway hyper-reactivity and airway inflammation (60). The observed increase in *Akkermansia muciniphila* within the lung microbiome of the Ova-res group, when compared to Ova-veh, suggested that RES could promote the growth of this potentially beneficial microorganism, which in turn, enhances mucous degradation and clearing of excess mucus, which was possibly responsible for the alleviation of asthma. In this context, inoculation of commensal *Escherichia coli* species was shown to attenuate OVA-induced airway inflammation in mice (61). We also noted in the current study using immunofluorescent staining that the production of epithelial MUC5ac protein was significantly increased in the lungs of OVA-veh-challenged mice when compared to the naïve mice, while RES was able to significantly decrease MUC5ac expression. The important relationship between lung microbiota and epithelial cell tight junction molecules highlighted the importance of our findings, indicating that RES treatment improved tight junctions, thereby interfering with exposure of sub-epithelial layer to microbial antigens.

RES also significantly modulated gut microbiota in OVA-treated mice. Specifically, RES significantly increased the abundance of the phylum Bacteroidetes, order Bacteroidales and species *Bacteroides acidifaciens.* Previous studies showed that a maternal diet rich in fibers indigestible to the host significantly increased the abundance of probiotic organisms, namely *Bacteroides acidifaciens*, which fermented fibers and produced SCFAs. SCFAs have been shown to induce the expression of transcription factor Foxp3 in T lymphocytes, leading to the induction of Tregs which in turn can drive the immune response towards tolerance and attenuate asthma (62, 63). Mechanistically, SCFAs induce Tregs by inhibiting histone deacetylase enzymes (HDACs) near the promoter region of the Treg master transcription factor, Foxp3 gene (64). In the current study, we found that resveratrol caused significant increase in butyrate in the colon of Ova-treated mice. Butyrate is derived from the fermentation non-digestible dietary fiber by commensal bacteria. Several studies have shown that butyrate can suppress asthma (32, 65). The butyrate produced in the large intestine can enter the circulation and mediate anti-inflammatory properties in the lungs (32). Mice fed with butyrate mice were shown to exhibit less severe allergic asthma (66). Thus, it is likely that the increased induction of butyrate by resveratrol in the colon of Ova-treated mice may contribute to attenuation of asthma.

LPS a TLR4 ligand, is the main component of the cell walls of gram-negative bacteria, that is released into the surrounding environment which induces a strong immune response through activation of NF-κB signaling pathway (67). Alveolar epithelial cells play a critical role in lung homeostasis and oxygen exchange. Alveolar epithelial type II (ATII) cells composed of about 5-7% only of the alveolar cell components are responsible for the production of surfactant proteins and some antimicrobial peptides (66, 68, 69). ATII cells are referred to as defenders of alveolus (69). Thus, LPS-induced injury of ATII cells could modulate the production of surfactant proteins and induce inflammatory responses through activation of TLR4 (70–74). LPS also induces the gene dysregulation of different tight junction proteins and thus damages the barrier function (75–77). It has been reported that RES can inhibit TLR4/NF-κB signaling cascade and thus alleviate LPS-mediated acute liver and lung inflammation in rats (78). In the current study, we used the OVA-mediated asthma mouse model to evaluate the effects of RES on the levels of LPS in the BALF and LPS metabolism, associated with gut and lung microbial dysbiosis. Our studies demonstrated that RES decreased the LPS levels in the BALF, and inhibited LPS biosynthesis. Furthermore, our *in vitro* investigation using murine lung epithelial cell line MLE-15 also discovered the role of RES in LPS-mediated alterations in the integrity and function of epithelial cells. Exposing monolayer of alveolar epithelial cells to LPS was enough to trigger barrier dysfunction and RES recovered integrity and functions of the epithelial cell monolayer. Our studies demonstrated that RES inhibited LPS-induced inflammation and maintained the integrity and barrier functions of the epithelial cells. In the current study, we found that LPS levels in the BALF in Ova-res group were significantly decreased, and while this can be explained by the resveratrol directly affecting LPS metabolism, it is also possible that resveratrol may cause changes in bacterial composition leading to decreased levels of LPS. For example, we found that resveratrol-treated mice showed increased levels of *Akkermansia muciniphila*, which are known to decrease intestinal permeability and lower LPS levels in circulation (79).

Flavonoids, which are polyphenolic plant secondary metabolites widely found in vegetables and fruits have been shown to suppress allergic response as well as suppress airway inflammation (80, 81). Polyphenols act through multiple pathways to suppress allergic asthma including suppression of inflammatory cytokines (82, 83), reversal of airway bronchoconstriction and bronchial hyper-reactivity (82, 84), downregulation of NF-κB activity (85), attenuation of oxidative stress (86), and promotion of healthy gut microbiota (87). However, there are no previous studies on polyphenols that have comprehensively investigated a combination of such pathways, including the gut-lung axis, as reported in the current study.

In summary, the current study showed that RES ameliorated the clinical parameters of asthma and promoted recovery to normalcy in the tissue architecture of the lung mucosa. The beneficial effects of RES could be due to the modulation of both lungs and gut microbial species. Particularly, RES promoted the growth of *Akkermansia mucinuphila* in the lungs and *Bacteroides acidifaciens* in the gut of OVA-challenged mice. As a mucous degrader, we believed that the presence of *Akkermansia muciniphila* in the lung tissue was able to improve parameters of lung function through the alleviation of excessive mucous production, as well as by driving epithelial integrity. In the gut, *Bacteroides acidifaciens* outgrowth could help establish an immunotolerant environment through the production of SCFAs, which is known to promote the generation of regulatory T cells (88). Importantly, RES could regulate lung and gut microbiota, and decrease LPS biosynthesis and thus contribute towards attenuation of asthma. This study suggested the use of RES, and perhaps other natural compounds, as potential novel therapeutics for the management of often incurable and costly inflammatory conditions, such as asthma.

## Data Availability Statement

The datasets presented in this study can be found in online repositories. The names of the repository/repositories and accession number(s) can be found below: https://www.ncbi.nlm.nih.gov/geo; GSE193246.

## Ethics Statement

The animal study was reviewed and approved by American Association for the Accreditation of Laboratory Animal Care (AAALAC)-accredited animal resource facility at the University of South Carolina, School of Medicine, Columbia, SC.

## Author Contributions

Conceptualization, MN and PN. Methodology, EA and HA. Resources, MN and PN. Experimentation, EA, HA, AM, and JZ. Writing—original draft preparation, EA and HA. Review and editing: PN and MN. Data interpretation, EA, HA, PN, and MN. All authors have read and agreed to the published version of the manuscript.

## Funding

This research was funded by the National Institutes of Health (NIH): P01AT003961, P20GM103641, R01AI129788, R01AI123947, R01AI160896 and R01ES030144.

## Conflict of Interest

The authors declare that the research was conducted in the absence of any commercial or financial relationships that could be construed as a potential conflict of interest.

## Publisher’s Note

All claims expressed in this article are solely those of the authors and do not necessarily represent those of their affiliated organizations, or those of the publisher, the editors and the reviewers. Any product that may be evaluated in this article, or claim that may be made by its manufacturer, is not guaranteed or endorsed by the publisher.

## Supplementary Material

Supplementary Figure 1
*Effect of resveratrol on physiological asthmatic features in the lungs of OVA-administered mice.* BALB/c mice were initially sensitized to OVA and treated with the Vehicle or RES as described in legend. To study the pulmonary functions, mice were chosen randomly and placed in one of 4 chambers of Buxco instrument. Pulmonary function tests shown include: changes in patterns of specific airway conductance (sGaw), peak of expiratory flow (PEF), time to inhale (Ti) and time to exhale (Te) among naïve, Ova-veh and Ova-res group as baseline response (green arrow), PBS exposure (red arrow) and 25 mg/ml methacholine (Mch) exposure (red arrow) **(A)**. Bar graphs show statistical comparison of sGaw, PEF, time to inhale and Te among naïve, Ova-veh and Ova-res group at the baseline response **(B)** and after challenge with 25 mg/mL methacholine **(C)**. Vertical bars in **(B, C)** represent Mean+/-SEM data from groups of 5 mice.

## Abbreviations

RES, resveratrol; OVA, ova-albumin; BALF, broncheoalveolar lavage fluid; F, frequency; MV, mean volume; sRaw, specific airway resistance; dT, delay time; Tregs, T-regulatory cells; LPS, lipopolysaccharide.
