# Supplementary figures and images for: The Ability of Resveratrol to Attenuate Ovalbumin-Mediated Allergic Asthma Is Associated With Changes in Microbiota Involving the Gut-Lung Axis, Enhanced Barrier Function and Decreased Inflammation in the Lungs
Source: Front Immunol. 2022 Feb 21;13:805770. doi: 10.3389/fimmu.2022.805770 (PMC8898895; doi:10.3389/fimmu.2022.805770)

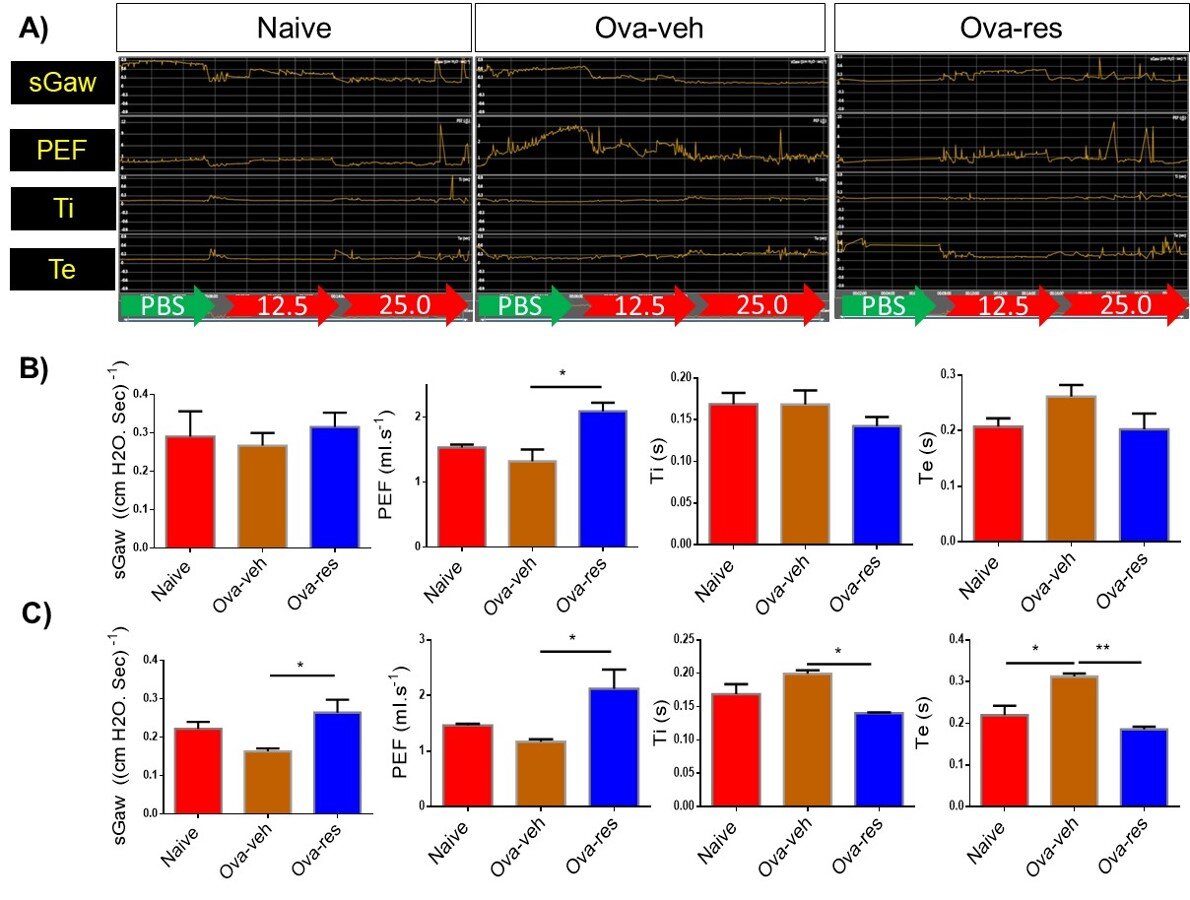

Supplement: Supplementary Figure 1 — Effect of resveratrol on physiological asthmatic features in the lungs of OVA-administered mice. BALB/c mice were initially sensitized to OVA and treated with the Vehicle or RES as described in legend. To study the pulmonary functions, mice were chosen randomly and placed in one of 4 chambers of Buxco instrument. Pulmonary function tests shown include: changes in patterns of specific airway conductance (sGaw), peak of expiratory flow (PEF), time to inhale (Ti) and time to exhale (Te) among naïve, Ova-veh and Ova-res group as baseline response (green arrow), PBS exposure (red arrow) and 25 mg/ml methacholine (Mch) exposure (red arrow) (A). Bar graphs show statistical comparison of sGaw, PEF, time to inhale and Te among naïve, Ova-veh and Ova-res group at the baseline response (B) and after challenge with 25 mg/mL methacholine (C). Vertical bars in (B, C) represent Mean+/-SEM data from groups of 5 mice. [file Image_1.jpg]
