# Supplementary material for: The Ability of Resveratrol to Attenuate Ovalbumin-Mediated Allergic Asthma Is Associated With Changes in Microbiota Involving the Gut-Lung Axis, Enhanced Barrier Function and Decreased Inflammation in the Lungs
Source: Front Immunol. 2022 Feb 21;13:805770. doi: 10.3389/fimmu.2022.805770 (PMC8898895; doi:10.3389/fimmu.2022.805770)
Supplement: Supplementary file 2 [file Table_1.docx]

**Table 1** Primer sequences for RT-qPCR analysis of tight junction protein and PPAR-γ genes in lung epithelial cells

| **Primer** | **Sequence** |
| --- | --- |
| **E-Cadherin (Forward)** | 5`-CAGGTCTCCTCATGGCTTTGC-3` |
| **E-Cadherin (Reverse)** | 5`-CTTCCGAAAAGAAGGCTGTCC-3` |
| **Claudin-18 (Forward)** | 5`- CCGCCGTGTTCCAGTATGAAG-3` |
| **Claudin-18 (Reverse)** | 5`-CGATCATCAGGGCTCGTACAG-3` |
| **Occludin (Forward)** | 5’-TTGAAAGTCCACCTCCTTACAGA-3’ |
| **Occludin (Reverse)** | 5`-CCGGATAAAAAGAGTACGCTGG-3` |
| **ZO-1 (Forward)** | 5`-GCCGCTAAGAGCACAGCAA-3 |
| **ZO-1 (Reverse)** | 5`-TCCCCACTCTGAAAATGAGGA-3` |
| **PPAR-γ (Forward)** | 5`-TCGCTGATGCACTGCCTATG-3 |
| **PPAR-γ (Reverse)** | 5`-GAGAGGTCCACAGAGCTGATT-3 |
| **GAPDH (Forward)** | 5`-AGGTCGGTGTGAACGGATTTG-3` |
| **GAPDH (Reverse)** | 5`-TGTAGACCATGTAGTTGAGGTCA-3` |
